# Comparative study between Salkowski reagent and chromatographic method for auxins quantification from bacterial production

**DOI:** 10.3389/fpls.2024.1378079

**Published:** 2024-06-11

**Authors:** Beatriz G. Guardado-Fierros, Diego A. Tuesta-Popolizio, Miguel A. Lorenzo-Santiago, Jacobo Rodriguez-Campos, Silvia M. Contreras-Ramos

**Affiliations:** ^1^Unidad de Tecnología Ambiental, Centro de Investigación y Asistencia en Tecnología y Diseño del Estado de Jalisco A.C. (CIATEJ), Guadalajara, Jalisco, Mexico; ^2^Unidad de Servicios Analíticos y Metrológicos, CIATEJ, Guadalajara, Jalisco, Mexico

**Keywords:** Salkowski, chromatography, PGPB, IAA, IBA, IPA

## Abstract

**Introduction:**

The Salkowski reagent method is a colorimetric technique used to determine auxin production, specifically as indole-3-acetic acid (IAA). It was developed to determine indoles rapidly; however, it does not follow Beer's law at high concentrations of IAA. Thus, there could be an overestimation of IAA with the Salkowski technique due to the detection of other indole compounds.

**Methods:**

This study aims to compare the Salkowski colorimetric method versus a chromatographic method to evidence the imprecision or overestimation obtained when auxins, such as indole-acetic acid (IAA), are determined as traits from promoting growth plant bacteria (PGPB), using ten different strains from three different isolation sources. The analysis used the same bacterial culture to compare the Salkowski colorimetric and chromatographic results. Each bacterium was cultivated in the modified TSA without or with tryptophan for 96 h. The same supernatant culture was used in both methods: Salkowski reagent and ultra-performance liquid chromatography coupled with a Mass Spectrometer (LC-MS/MS).

**Results:**

The first method indicated 5.4 to 27.4 mg L^-1^ without tryptophan in ten evaluated strains. When tryptophan was used as an inductor of auxin production, an increase was observed with an interval from 4.4 to 160 mg L^-1^. The principal auxin produced by all strains was IAA from that evaluated by the LC-MS/MS method, with significantly higher concentration with tryptophan addition than without. Strains belonging to the *Kocuria* genus were highlighted by high IAA production. The indole-3-propionic acid (IPA) was detected in all the bacterial cultures without tryptophan and only in *K. turfanensis* As05 with tryptophan, while it was not detected in other strains. In addition, indole-3-butyric acid (IBA) was detected at trace levels (13-16 µg L^-1^).

**Conclusions:**

The Salkowski reagent overestimates the IAA concentration with an interval of 41-1042 folds without tryptophan and 7-16330 folds with tryptophan as inductor. In future works, it will be necessary to determine IAA or other auxins using more suitable sensitive techniques and methodologies.

## 1 Introduction

Auxins are defined as “a group of compounds with similar growth-regulating action described in plants”, known as phytohormones to be the first discovered in plants, such as indole-3-acetic acid (IAA), phenylacetic acid, or indole-3-butyric acid (IBA) (Sheldrake, 2021; Bogaert et al., 2022). Auxin has been reported in different development processes, such as cell growth expansion to drive embryogenesis, differentiation, and tissue patterning (Sheldrake, 2021; Bogaert et al., 2022). Due to all these roles, they have been used in different agricultural applications and will be natural, synthetic, or precursors (Morffy and Strader, 2020). The most researched in plants is indole-3-acetic acid (IAA), present in young growing cells in shoot tips and leaves and in developing flowers, seeds, and roots (Sheldrake, 2021).

**Figure 1:**
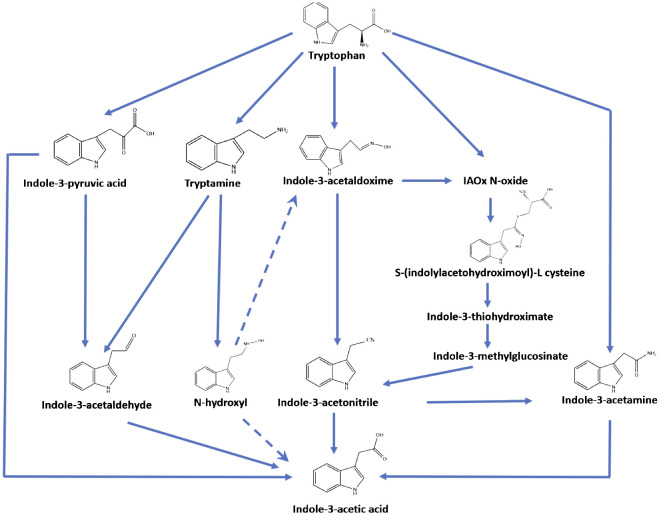
Bacteria, fungi, plants, and algae reported four primary biosynthetic ways for IAA production from tryptophan—own elaboration and modified from Labeeuw et al. (2016).

Auxins are synthesized in different organisms, such as animals, plants, green algae, bacteria, and fungi (Morffy and Strader, 2020; Sheldrake, 2021; Bogaert et al., 2022). They share some intermediaries’ compounds in the biosynthesis pathway. However, some steps have not yet been elucidated for all organisms (**Figure 1**). Tryptophan is a substrate generalized in the first step of the pathway. However, tryptophan aminotransferases (TAA) are non-specific, and some authors have demonstrated that they work with other aromatic amino acids (phenylalanine, tyrosine, and tryptophan) and others such as leucine, alanine, methionine, and glycine (Kittell et al., 1989; Tao et al., 2008; Sheldrake, 2021).

This biosynthetic pathway has been presented into four main groups: (1) indole acetamide (IAM) way by oxidation through an oxygenase; (2) indole-3-pyruvic acid (IPA) way by an aminotransferase; (3) tryptamine (TRY) way through a tryptophan decarboxylase, and (4) indole-3-acetonitrile (IAN) way by hydrolyzation on the amino group (Morffy and Strader, 2020; Lin et al., 2022). The indole-3-pyruvic acid (IPA) pathway was reported widely in many bacteria (benefic and phytopathogens) and some cyanobacteria (Lin et al., 2022).

The Salkowski reagent method is a colorimetric technique used to determine auxin production, specifically indole-3-acetic acid (IAA), in different samples, including bacterial strains, rhizobacteria, and plant tissues (Mayer, 1958; Ehmann, 1977; Glickmann and Dessaux, 1995; Gilbert et al., 2018; Nascimento et al., 2019). This method was first developed by Gordon and Weber (Gordon and Weber, 1951) to determine indoles rapidly. Their method was modified from earlier (Mitchell and Brunstetter, 1939; Tang and Bonner, 1948). Later methods used sulfuric acid, a higher time for reaction, or hydrogen peroxide, but they had unstable coloration. So, Gordon and Weber (1951) proposed the optima Salkowski reagent procedure by estimating the IAA and used in the actuality: 1 mL of the sample mixed with 2 mL of the Salkowski reagent (1 mL 0.5 M FeCl_3_, 50 mL 35% HClO_4_), incubation for 25 minutes in the dark at room temperature. Absorbance is measured in a spectrophotometer at a wavelength fixed at 530 nm. This method was established with an optimal interval of 0.2 to 0.45 μg L^-1,^ and they stated that Beer’s law is not followed at high concentrations of IAA. In addition, the Salkowski technique and different variants have been reported in the detection of IAA at low concentrations (0.5 to 20 μg mL^-1^) or higher concentrations (5 to 200 μg mL^-1^) (Glickmann and Dessaux, 1995). However, Gilbert et al. (2018) reported that IAA cannot be detected at ≤ 5 μg mL^-1^ when no L-tryptophan is added to the medium, and it cannot be detected at ≤ 10 μg mL^-1^ when L-tryptophan is supplemented (5 mM) due to a high background absorbance.

Different reports have indicated that the Salkowski test cannot be able to distinguish the indole compounds (Glickmann and Dessaux, 1995; Goswami et al., 2015; Gilbert et al., 2018); however, it is a recurrent technique for auxins determination in bacteria. So, an overestimation of IAA could be detected with the Salkowski technique due to the detection of other indole compounds. Also, IPA could be synthesized by various bacteria as the result of nonspecific transamination from tryptophan, and yet bacteria producing low amounts of IPA could, therefore, be misidentified as auxin producers by the Salkowski technique (Kittell et al., 1989; Glickmann and Dessaux, 1995).

Despite its limitations, the Salkowski reagent method is widely used for detecting IAA production in different studies or for determining the traits in promoting growth plants from microorganisms (PGPM) such as bacteria, fungi, and phytopathogenic bacteria (Goswami et al., 2015; Devi et al., 2022).

Alternative methods for determining auxin production or indole acid compounds have been reported, such as adaptations for determining small molecules using the enzyme-linked immunosorbent assay method (ELISA), which is frequently used for proteins (Hayat et al., 2020), as well as more precise methods including mass spectrometry-based metabolomics, gas chromatography-mass spectrometry (GC-MS), high-performance thin-layer chromatography (HPTLC), high-performance liquid chromatography (HPLC) and different variants or detectors (Badenoch-Jones et al., 1982; Wikoff et al., 2009; Lee et al., 2010; Goswami et al., 2015; Pedras C. and Yaya, 2015; Arund et al., 2016).

In some cases, ELISA assays have been used with mycorrhizal fungi, where studies have shown that auxin plays a crucial role in regulating the development of arbuscular mycorrhizal symbiosis (Liao et al., 2018). ELISA assays can quantify auxin levels and assess their impact on symbiotic interactions between plants and mycorrhizal fungi (Shaul-Keinan et al., 2002). It could detect auxin production in bacterial cultures as a first step. However, the IAA samples must be methylated by diazomethane before ELISA analysis. So other auxins and derivates methylated can rection with the antibodies, such as Indole-3-acetyl-L-alanine, Indol-3-propionic acid, 4-Chloroindole-3-acetic acid, α-Naphthyl acetic acid, among other, according with ELISA kit’s provider (Agrisera AB™, Vännäs, Sweden). So, ELISA assays have limitations, and no precision of IAA or specific auxins could be determined.

Chromatographic methods provide a more specific and accurate quantification of IAA than the spectrophotometric method using the Salkowski reagent. However, they could be expensive or difficult to access for research, and it is not a quick determination.

So, this study aims to compare the Salkowski colorimetric method versus a chromatographic method to evidence the imprecision or overestimation obtained when auxins, such as indole-acetic acid (IAA), are determined as traits from promoting growth plant bacteria (PGPB) using ten different strains from three different isolation sources. Also, tryptophan was used as an inductor-dependent auxin synthesis, and the results were compared without tryptophan addition.

## 2 Materials and methods

### 2.1 Biological material

Ten different bacteria were selected to determine the production of auxins in a liquid medium. These bacteria were isolated from different sources, previously identified by 16S, and deposited in the GenBank database (**Table 1**).

**Table 1:** Bacteria information: strain code, source isolation, and GenBank’s codes access numbers for 16S identification.

Code	Strain	Source isolation	GenBank accession
As05	*Kocuria turfanensis*	Air	OP934049
As18	*Staphylococcus equorum*	Air	OP934050
As25	*Kocuria sediminis*	Air	OP934051
As32	*Kocuria turfanensis*	Air	OP934052
As33	*Rhodococcus rhodochrous*	Air	OP934053
As38	*Rhodococcus rhodochrous*	Air	OP934054
As41	*Kocuria turfanensis*	Air	OP934055
9–3	*Pseudomonas koreensis*	Agricultural waste	OM966650
19–1	*Pseudomonas fluorescens*	Field agricultural soil	OM966651
25–1	*Pseudomonas poae*	Field agricultural soil	OM966652

Air bacteria were isolated from air samples of 100 L each were collected from the metropolitan area of Guadalajara, Mexico, at two sites: “San Juan de Dios” (20° 40’31” N; 103° 20’24” W) and “Las Pintas” (20° 34’35” N; 103° 19’32” W). These areas are highly populated; San Juan de Dios is in the downtown area of Guadalajara and hosts one of the busiest markets in the city, while Las Pintas is an industrial zone. Sampling was conducted using an air sampler gun (Millipore^®^ M air T, U.S.A.) in triplicate, and the samples were spread on Luria-Bertani agar (LB, for bacteria) plates. Some bacteria were isolated from an agricultural waste mixture such as worm casting, whey, molasses, cow manure, and field agricultural soil from Zapotlanejo, Jalisco, Mexico (21° 0’ 48.69” N; 103° 12’ 11.84” W). One hundred grams of waste or soil (20 cm from topsoil) were sampled in plastic bags, transported to the laboratory at 4°C and processed to isolation bacteria by serial dilution method (Tuesta-Popolizio et al., 2021) on medium modified with tryptone soy agar (TSA): C_6_H_12_O_6_ 5 g L^-1^; K_2_HPO_4_ 1 g L^-1^, (NH_4_) NO_3_ 0.4 g L^-1^, NaCl 0.2 g L^-1^, MgSO_4_·7H_2_O 0.4 g L^-1^, Tryptone 20 g L^-1^.

The FastDNA Spin Kit (MP Biomedicals, USA) for Soil was utilized for DNA extraction. DNA concentration was assessed using UV-VIS spectrophotometry (NanoDrop-2000, Thermo Scientific, USA) and stored at -20°C until use. Sequencing was conducted at Sanger PSOMAGEN INC. (USA) using primers 27F and 1492R for amplified sequencing (Osborne et al., 2005). Sequences were manually checked and trimmed using Sequencher 5.4.6 (Gene Codes Corporation). The final sequences were compared against the NCBI 16S (BLAST) and deposited in the GenBank database. (http://www.ncbi.nlm.nih.gov/BLAST) (**Table 1**).

### 2.2 Auxins determination

The auxins analysis used the same bacterial culture to compare the Salkowski colorimetric and chromatographic results. Each bacterium was cultivated in the modified TSA described above.

Sixty-six tubes (50 mL) containing 10 mL of modified TSA medium were prepared, thirty-three were supplemented with L-tryptophan (T) (0.1 g L^-1^) (Sigma Aldrich ≥98%), and thirty-three were left without supplement. The L-tryptophan was added as an IAA precursor. One bacteria colony, taken from a TSA petri dish plate, was added to each tube in triplicate (n=3), and three tubes were used as the control medium (without inoculation). The tubes were incubated at 30 ± 2°C for 96 h. After incubation, cultures were centrifuged at 4,000 rpm for 10 minutes. Salkowski’s colorimetric and chromatographic method determined auxins in the supernatant after solvent extraction, as described below. The same supernatant culture was used in both methods.

#### 2.2.1 Salkowski colorimetric method

The original protocol of the Salkowski assay reported by Gordon and Weber was adapted for a 96-well plate format (Gordon and Weber, 1951; Gilbert et al., 2018). In a Corning 96-well transparent plate were placed 100 µL of each bacterial supernatant and 200 µL of Salkowski reagent were added: 0.5 M FeCl_3_, 97% reagent grade, and 34.3% perchloric acid, ACS grade). Samples were incubated with the Salkowski reagent at room temperature for 30 min in darkness; the appearance of an orange-pink color suggests the possible production of IAA, and the orange color indicates the presence of other indole compounds, and their absorbance was recorded at 530 nm in an xMark™ microplate spectrophotometer (BIO-RAD, USA). The amount of auxin in the bacteria cultures was estimated with a calibration curve using the IAA standard (Sigma Aldrich ≥98% Part No I3750–5G) in the 0–250 mg L^-1^ range.

#### 2.2.2 Extraction and quantification of auxins by LC-MS/MS

The extraction of auxins was carried out using the modified method by Goswami et al. (2015). Each culture supernatants were acidified to pH 2.85 using HCL 1 N. 2.5 mL of culture was extracted by auxin with two consecutive extractions using ethyl acetate (1:2 v:v) and stirred vigorously for 1 minute in the vortex. A subsample (2 mL) of ethyl acetate fraction was separated, evaporated dryness under a speed vac concentrator (vacufuge^®^, Eppendorf, Germany), and dissolved in 2 mL of methanol. This extract was filtered by 0.22 µm and analyzed by an ultra-performance liquid chromatography coupled with a Mass Spectrometer (LC-MS/MS) analysis.

The identification and quantification of phytohormones were performed using An Acquity I-class UPLC system (Waters, Milford, MA, USA) equipped with a Waters ACQUITY UPLC^®^ CSH C18 column (1.7 µm, 2.1 × 50 mm). They were coupled to a Mass Spectrometer Xevo^®^ TQs triple quadrupole (Waters MS Technologies, Manchester, UK). The mobile phases were 0.1% formic acid (A) and methanol (B), with a flow rate of 0.25 mL min^-1^. The column temperature was maintained at 40°C, and the elution gradient was 90% A as the initial condition, 50% A at 1 min, 20% A at 5 min, and 90% A at 6 min. The injection volume was 3 µL. The conditions of the Mass Spectrometer were 2.5 kV capillary voltage, the desolvation temperature was 500°C, and the desolvation gas flow was set at 1000 L/h. The ionization source was in electrospray positive mode, and the temperature was 150°C. Measurements were performed in multiple reaction monitoring (MRM) modes (**Table 2**). Concentrations were obtained using a standard curve prepared from 5 to 500 µg L^-1^, with standards (purity > 99.0%) of the IAA (indole-3-acetic acid), IBA (indole-3-butyric acid), and IPA (indole-3-propionic acid) (Sigma-Aldrich^®^ Parts No I3750–5G, 57310–5G, 57400–5G, respectively). MassLynx and TargetLynx Software were used to process all data.

**Table 2:** Precursor ions and selected transitions for each auxin in the LC-MS/MS method.

Compound	Retention time (min)	Precursor ion (*m/z*)	Product (*m/z*)	Cone (V)	Collusion energy (V)
IAA*	2.17	176.15	103.1 130.15	10 10	30 20
IPA*	2.47	190.2	130.15 172.15	30 30	20 13
IBA*	2.79	204.2	130.15 186.15	10 10	25 15

*IAA, Indole-3-acetic acid; IBA, Indole-3-butyric acid; IPA, Indole-3-propionic acid.

### 2.3 Statistical analysis

All data were previously subjected to normality tests to compare significance differences between strains evaluated with ANOVA, a *post hoc* Tukey test, and a 95% significance level. All data were analyzed using Statgraphics software, version Centurion XVI.

## 3 Results

### 3.1 Salkowski method

**Figure 2:**
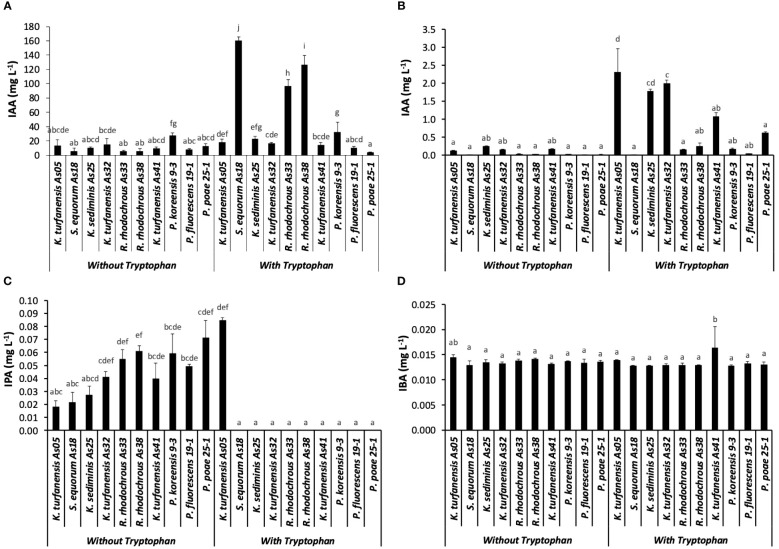
Auxins concentration from ten strains, without and with tryptophan, **(A)** Salkowski method represented as IAA; **(B)** IAA, **(C)** IPA, and **(D)** IBA determined by LC-MS/MS, IPA. Bars indicate the standard deviation for n=3. The different lowercase letters indicate significant differences between strains.

Auxin concentrations [indole-3-acetic acid (IAA)] by the Salkowski method were detected from 5.4 to 27.4 mg L^-1^ without tryptophan in ten evaluated strains. When tryptophan was used as an inductor of auxin production, an interval from 4.4 to 160 mg L^-1^ was observed (**Figure 2A**). *P. koreensis* 9–3 presented the highest IAA concentration (27.45) without tryptophan, determined by Salkowski reagent, followed by *K. turfanensis* As32, As05, and *K. sediminis* As25 (14.88, 13.73, and 10.21 mg L^-1^ respectively) (p < 0.05). Significantly low concentrations were detected in *R. rhodochrous* As33, As38, and *S*. *equorum* As18 (< 6 mg L^-1^) without tryptophan. Almost all IAA concentrations found with tryptophan were higher than without tryptophan. An increase was observed with the auxin’s inductor from 0.35 folds (*P. poae* 25–2) to 27.5 folds (*S*. *equorum* As18). The strains *S*. *equorum* As18, *R. rhodochrous* As38, and As33 showed significantly higher IAA concentrations (160, 126, and 96 mg L^-1,^ respectively) than other strains when supplemented with tryptophan (p < 0.05).

### 3.2 LC-MS/MS method

The principal auxin produced by all strains was IAA from that evaluated by the LC-MS/MS method, with significantly higher concentration with tryptophan addition than without (p < 0.05). The highest concentration was observed in *K*. *turfanensis* As05 and AS32 strains (2.31 and 2.0 mg L^-1^), followed by *K*. *sediminis* As25 (1.78 mg L^-1^) than other strains (p < 0.05). These values contrast with the lowest concentration detected in *S. equorum* AS18 (0.001 mg L^-1^) and *P. fluorescens* 19–1 (0.04 mg L^-1^) (**Figure 2B**). The IAA concentrations without tryptophan were from 0.01 to 0.25 mg L^-1^ without significant differences between the strains (p ≥.0.05). *K. sediminis As25* and *K. turfanensis* As41 show a higher value than other strains (p ≥.0.05). Strains belong to the *Kocuria* genus, highlighted by high IAA production.

**Figure 3:**
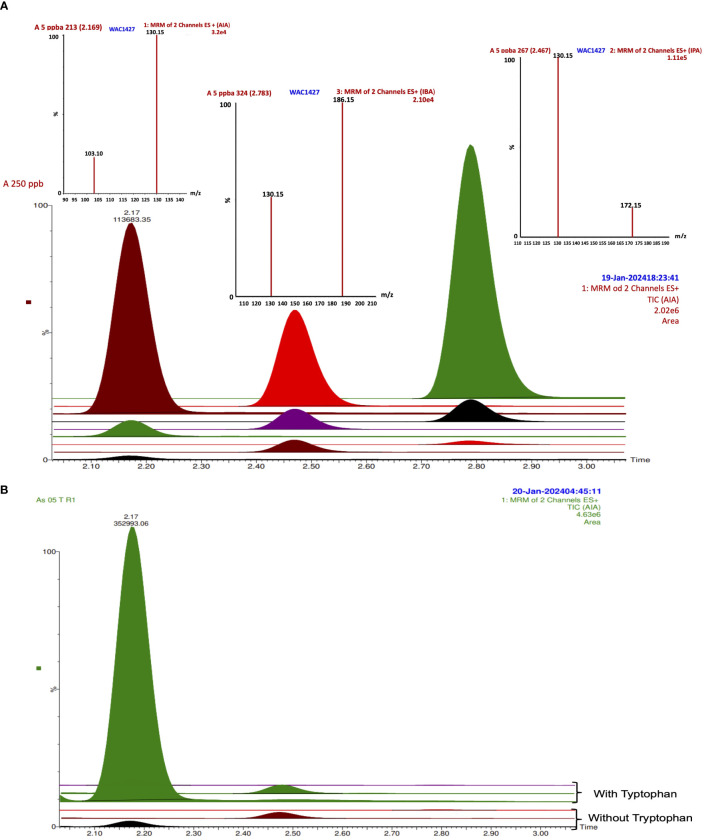
IAA, IPA, and IBA TIC and spectrums by LC-MS/MS in **(A)** standard curve from 5 to 250 ppb; **(B)** TIC from strain As05 without and with tryptophan.

The indole-3-propionic acid (IPA) was detected in all the bacterial cultures without tryptophan, and only in *K. turfanensis* As05 with tryptophan, while in other strains, it was not detected (**Figure 2C**). No significant differences were observed between the strains (p ≥.0.05). Although some strains presented high values compared with others, for example, *K. turfanensis* As05 with tryptophan (0.085 mg L^-1^), followed by *P. poae* 25–1 (0.072 mg L^-1^), *R. rhodochrous* As38 and As33 (0.061 and 0.055 mg L^-1^ respectively) without significant differences between them (p ≥.0.05). So, this auxin was detected in the 20–85 µg L-1 range with a perfectly defined chromatogram peak and verified with retention time and transitions (**Figures 3A, B**; **Table 2**). These results indicated the high sensibility of chromatographic methods.

In addition, indole-3-butyric acid (IBA) was detected to trace levels (13–16 µg L^-1^) with a consistent detection into the interval of the standard curve of work (**Figures 2D**, **3A**). Only *K. turfanensis* As41 supplemented with tryptophan presented significantly higher IBA concentration than other strains. No significant differences were observed between strains growing with or without tryptophan (p ≥.0.05).

These results highlight the overestimation of IAA by the Salkowski reagent in the strains evaluated in this work. **Table 3** shows overestimation values for each strain, considering only IAA concentration in both techniques. An interval of 41–1042 overestimation folds without tryptophan and 7–16330 folds with tryptophan were detected. No other auxins (IPA and IBA) were considered for this assessment.

**Table 3:** Overestimation of IAA comparing Salkowski reagent concentration and IAA chromatographic concentration.

Condition	Strain	Overestimation of IAA*
**Without Tryptophan**	*K. turfanensis As05*	102.08
	*S. equorum As18*	594.71
	*K. sediminis As25*	41.55
	*K. turfanensis As32*	90.45
	*R. rhodochrous As33*	161.99
	*R. rhodochrous As38*	187.29
	*K. turfanensis As41*	55.82
	*P. koreensis 9–3*	1042.53
	*P. fluorescens 19–1*	502.59
	*P. poae 25–1*	719.38
**With Tryptophan**	*K. turfanensis As05*	7.84
	*S. equorum As18*	16330.25
	*K. sediminis As25*	13.07
	*K. turfanensis As32*	8.45
	*R. rhodochrous As33*	624.33
	*R. rhodochrous As38*	506.46
	*K. turfanensis As41*	13.43
	*P. koreensis 9–3*	178.74
	*P. fluorescens 19–1*	230.98
	*P. poae 25–1*	7.02

* IAA concentration was detected by the Salkowski reagent between the IAA concentration detected by LC-MS/MS.

## 4 Discussion

Chemically, the indoles are a heterocyclic compound group containing a benzene ring joined to a pyrrole ring, which could be chemically synthesized by different reactions (Li, 2005). Indole or indole-like compounds have been widely reported in the environment and organisms, such as plants, soil bacteria, gut microbiota, fungi, insects, and human cells. Auxins belong to the chemical indole group. Indole has a vital role in bacterial biofilm formation and virulence mechanism in different processes of pathogenic bacteria. Their production is well reported, including Gram-positive and Gram-negative bacteria through the enzymatic degradation of tryptophan (**Figure 1**), and different indole analogs are produced such as 3-methylindole (skatole), indoxyl sulfate, indole-3-propionic acid, indole-butyric acid (Martínez-Morales et al., 2003; Darkoh et al., 2015; Morffy and Strader, 2020; Lin et al., 2022).

The early version of the Salkowski reagent was developed to rapidly determine indole compounds (Tang and Bonner, 1948; Gordon and Weber, 1951). It was established in an optimal interval of 0.2 to 0.45 μg mL^-1,^ and Gordon and Weber (1951) stated that Beer’s law is not followed at high concentrations of IAA where the slope of the line tended to decrease. A similar behavior was detected in the experiment to observe different IAA standard curves for the Salkowski reaction (**Supplementary Figure S1**, **Supplementary Material**). In addition, the Salkowski technique and different variants have been reported in the detection of IAA at low concentrations (0.5 to 20 μg mL^-1^) or higher concentrations (5 to 200 μg mL^-1^) (Glickmann and Dessaux, 1995). Some authors reported IAA detected limits at ≥ 5 μg mL^-1^ when no L-tryptophan is added to the medium and at ≥ 10 μg mL^-1^ with L-tryptophan supplemented at 5 mM due to a high background absorbance (Gilbert et al., 2018).

**Figure 4:**
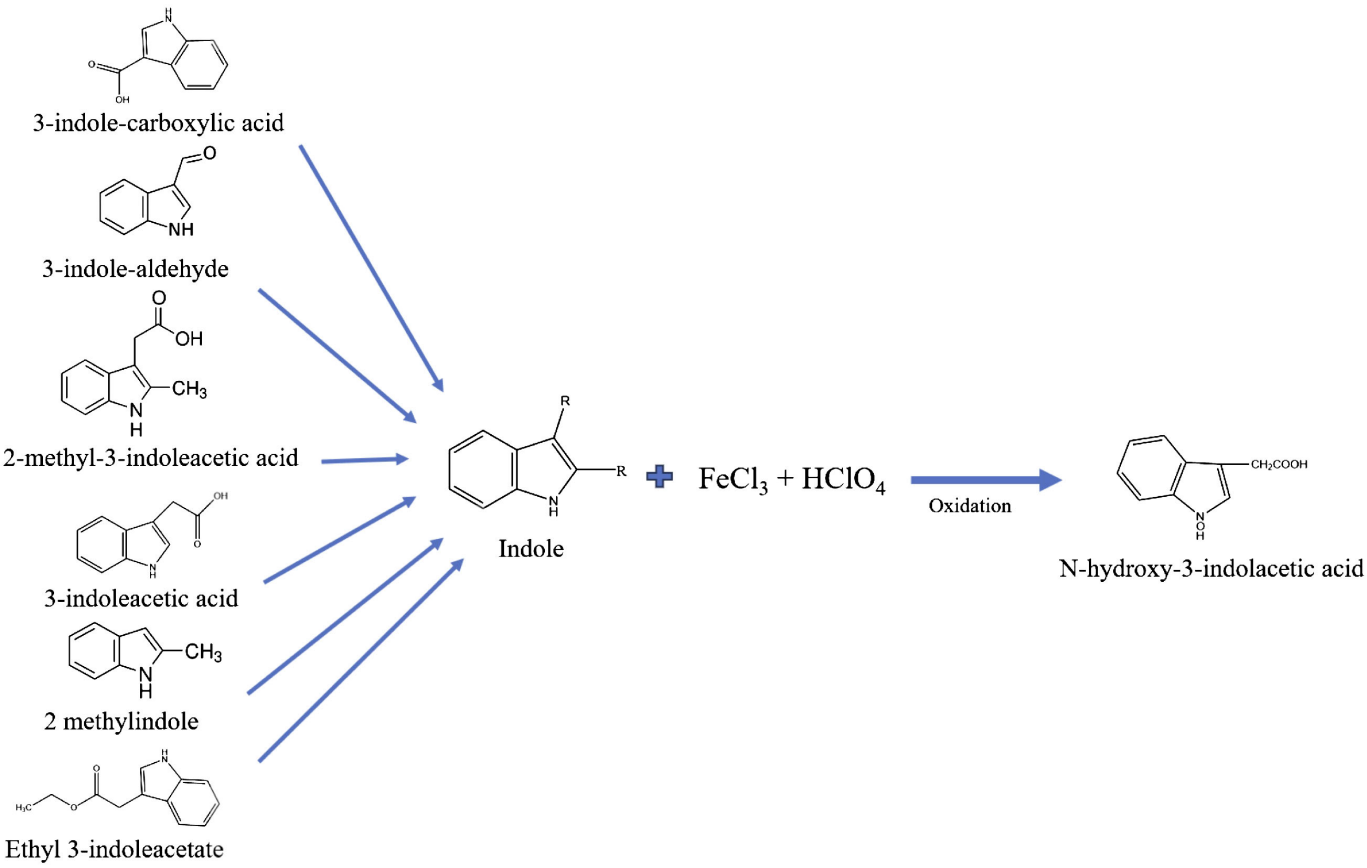
Salkowski reaction for indole compounds oxidation in the presence of Fe.

Salkowski reagent is not specific to IAA and reacts with other similar indole compounds (**Figure 4**) (Glickmann and Dessaux, 1995; Goswami et al., 2015; Gilbert et al., 2018). Early works indicated that the Salkowski reagent was less sensitive (10-fold) than the Ehrlich and van Urk reagent, with poor indol specificity (Ehmann, 1977). The fact is that Salkowski reacts not only with auxin (i.e., IAA) but also with indole pyruvic acid (IPA), indole acetamide (IAM), and other indole analog compounds, as is presented in **Figure 4**. Salkowski’s reaction is based on the reactivity of the nitrogen atom in the pyrrole ring, as was postulated by Hinsvark et al. (1953). They demonstrated that the colored product was an oxidation of IAA in the presence of Fe II, forming the N-hydroxy-3-indole-acetic acid product, which could be formed from any other indole analog compound and yield other indole derivated. Revelou et al. (2019) demonstrated that indole-3-acetic acid (IAA) and its metabolites in plants of the Brassicaceae family belong to IAA amide conjugates with amino acids bearing a free carboxylic group or a methyl ester group. This was achieved through high-resolution mass spectrometry (HRMS) and ultra-performance liquid chromatography quadrupole time-of-flight mass spectrometry (UPLC-QToF-MS) methodology.

Few studies have reported comparing the IAA estimations by spectrophotometric and chromatographic methods. Szkop and Bielawski (2013) reported a detection limit of IAA by reverse phase high-performance liquid chromatography (RP-HPLC) obtained from *P. putida* was below 0.015 μg mL^-1^ from a culture of 72 h.

Goswami et al. (2015) compared the estimation of the IAA production (By the Salkowski method) of 5 different strains of bacteria (*Pseudomonas aeruginosa*, *Kocuria turfanensis*, *Kocuria flava*, *Bacillus subtilis*, and *Bacillus licheniformis*) after 96 hours of growth with tryptophan. They obtained values from 4.230 to 37.6 mg L^-1^, while by HPTLC, they detected from 1.6 to 8 mg L^-1^. Tesese results show a difference between the estimates due to Salkowski’s reagent method and more precise analytical techniques such as High-Performance Thin Layer Chromatography (HPTLC).

The production of auxins by bacteria is a fact and has been widely documented, independent of the methodology for their detection. It is recognized as a mechanism by which plant growth-promoting bacteria (PGPB) can enhance plant growth and root development. Bacterial species, such as *Pseudomonas putida*, *Microbacterium testaceum*, *Pantoea agglomerans*, *Bacillus* spp., *Rhizobium* strains, and *Azospirillum brasilense* have been reported to produce auxins such as indole-3-acetic acid (IAA), 4-chloroindole-3-acetic acid, indole-3-butyric acid (IBA), indole-3-propionic acid (IPA) (Talboys et al., 2014; Luziatelli et al., 2020; Morffy and Strader, 2020; Lin et al., 2022; Tanveer and Ali, 2022; Yadav et al., 2022). All of them had tryptophan as an inductor. Tanveer and Ali (2022) tested 0, 200, and 500 μg mL^-1^ of tryptophan concentration, and they detected higher auxin production (41.4 and 24.8 µg mL^-1^) with 500 μg mL^-1^ tryptophan. The strains evaluated in our work presented lower IAA auxin concentration, with less tryptophan (100 μg mL^-1^).

Auxins from bacteria have positively affected host plants’ root system development and architecture (Yadav et al., 2022). In addition, bacterial auxins have demonstrated a relevant role in improving root growth and branching during micropropagation plants or *in vitro* assays (Luziatelli et al., 2020; Tzipilevich et al., 2021). Furthermore, the production of auxins by bacteria has been associated with the modulation of plant immune system activation, which is necessary for efficient interaction with auxin-secreting beneficial bacteria (Ali, 2019) and pathogen defense (Lin et al., 2022). Reports suggest that less than 1 mM of auxins promote, inhibit, or modify the growth and development of plants (Martínez-Morales et al., 2003).

Independently of the amount detected in some bacteria by the Salkoski method, PGPB bacteria represent a beneficial partner for the plants when interacting in their ecological niche. In future works, it will be necessary to determine IAA or other auxins with more suitable techniques and methodologies sensitive. However, analytical methodologies imply expensive infrastructures and equipment that are not always easily accessible.

## 5 Conclusions

The tens strain evaluated presented IAA production. However, the Salkowski reagent overestimates the concentration with an interval of 41–1042 overestimation folds without tryptophan and 7–16330 folds with tryptophan cultures. In addition, other auxins analogs were detected at trace levels (IPA and IBA). Independently of the IAA concentration detected in some bacteria by the Salkowski method, PGPB bacteria represent a beneficial partner for the plants when interacting in their ecological niche. In future works, it will be necessary to determine IAA or other auxins with more suitable techniques and methodologies sensitive. However, analytical methodologies imply expensive infrastructures and equipment that are not always easily accessible.

## Data availability statement

The original contributions presented in the study are included in the article/**Supplementary Material**, further inquiries can be directed to the corresponding author/s.

## Author contributions

BG: Formal analysis, Investigation, Writing – original draft. DT: Investigation, Methodology, Writing – original draft. ML: Data curation, Investigation, Writing – original draft. JR: Investigation, Methodology, Writing – original draft. SC: Formal analysis, Investigation, Supervision, Writing – review & editing.
