# Supplementary figures and images for: Comparative study between Salkowski reagent and chromatographic method for auxins quantification from bacterial production
Source: Front Plant Sci. 2024 Jun 11;15:1378079. doi: 10.3389/fpls.2024.1378079 (PMC11212217; doi:10.3389/fpls.2024.1378079)

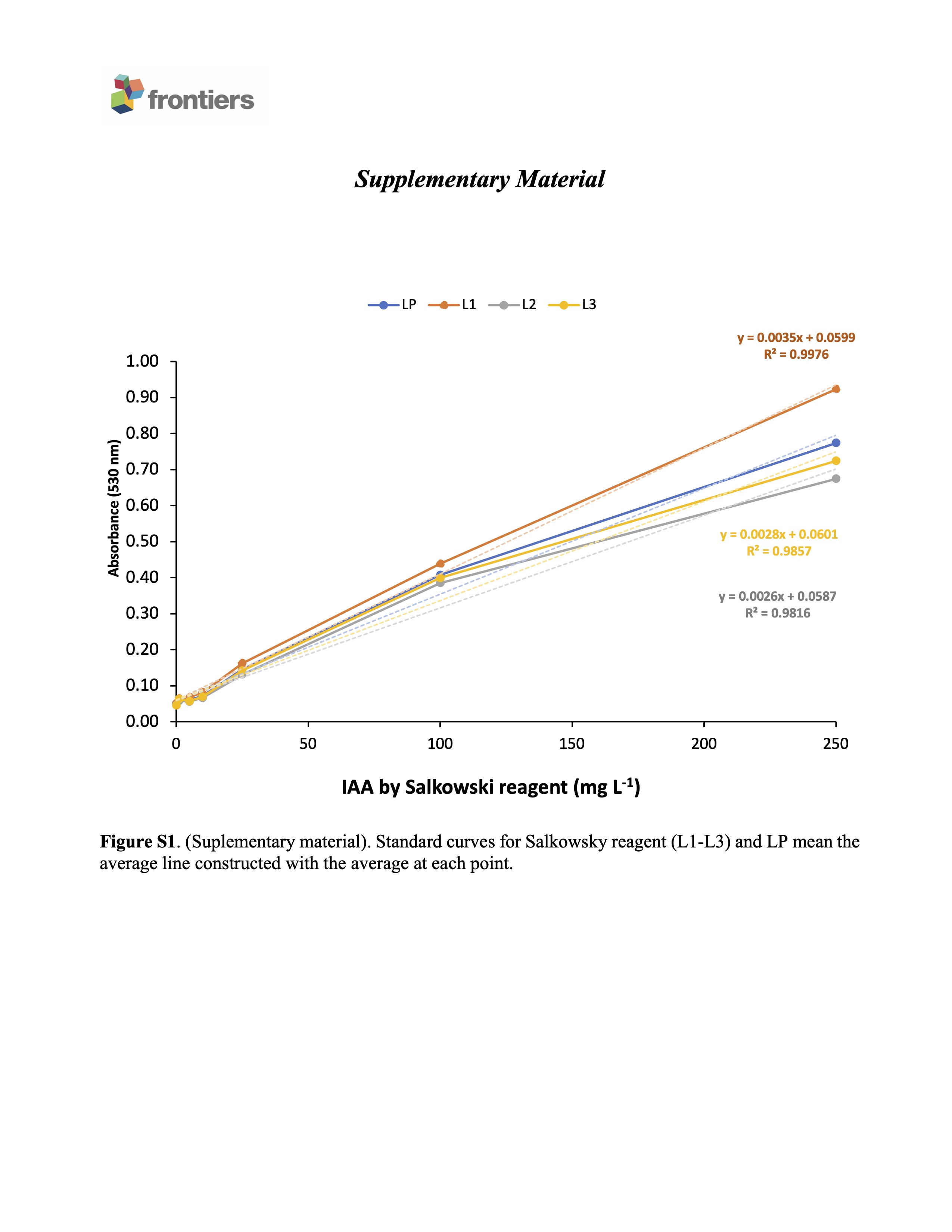

Supplement: Supplementary file 1 [file Image_1.jpeg]
